# A Hybrid Digital Parenting Program Delivered Within the Malaysian Preschool System: Protocol for a Feasibility Study of a Small-Scale Factorial Cluster Randomized Trial

**DOI:** 10.2196/55491

**Published:** 2024-04-26

**Authors:** Hal Cooper, Farah Zeehan Mohd Nadzri, Seema Vyas, Rumaya Juhari, Nellie Ismail, Zarinah Arshat, Durgesh Rajandiran, Laurie Markle, Francisco Calderon, Inge Vallance, G J Melendez-Torres, Chiara Facciolà, Vanisa Senesathith, Frances Gardner, Jamie M Lachman

**Affiliations:** 1 Department of Social Policy and Intervention University of Oxford Oxford United Kingdom; 2 Faculty of Human Ecology Universiti Putra Malaysia Selangor Malaysia; 3 Parenting for Lifelong Health Oxford United Kingdom; 4 The Medical School University of Exeter Exeter United Kingdom; 5 Innovations in Development, Education and the Mathematical Sciences International Reading United Kingdom; 6 Centre for Social Science Research University of Cape Town Cape Town South Africa

**Keywords:** parenting intervention, chatbot-led public health intervention, engagement, implementation science, feasibility, evidence-based program

## Abstract

**Background:**

The United Nations’ Sustainable Development Goal 4, and particularly target 4.2, which seeks to ensure that, by 2030, all children have access to quality early childhood development, care, and preprimary education so that they are ready for primary education, is far from being achieved. The COVID-19 pandemic compromised progress by disrupting education, reducing access to well-being resources, and increasing family violence. Evidence from low- and middle-income countries suggests that in-person parenting interventions are effective at improving child learning and preventing family violence. However, scaling up these programs is challenging because of resource constraints. Integrating digital and human-delivered intervention components is a potential solution to these challenges. There is a need to understand the feasibility and effectiveness of such interventions in low-resource settings.

**Objective:**

This study aims to determine the feasibility and effectiveness of a digital parenting program (called Naungan Kasih in Bahasa Melayu [Protection through Love]) delivered in Malaysia, with varying combinations of 2 components included to encourage engagement. The study is framed around the following objectives: (1) to determine the recruitment, retention, and engagement rates in each intervention condition; (2) to document implementation fidelity; (3) to explore program acceptability among key stakeholders; (4) to estimate intervention costs; and (5) to provide indications of the effectiveness of the 2 components.

**Methods:**

This 10-week factorial cluster randomized trial compares *ParentText*, a chatbot that delivers parenting and family violence prevention content to caregivers of preschool-aged children in combination with 2 engagement components: (1) a WhatsApp support group and (2) either 1 or 2 in-person sessions. The trial aims to recruit 160 primary and 160 secondary caregivers of children aged 4-6 years from 8 schools split equally across 2 locations: Kuala Lumpur and Negeri Sembilan. The primary outcomes concern the feasibility and acceptability of the intervention and its components, including recruitment, retention, and engagement. The effectiveness outcomes include caregiver parenting practices, mental health and relationship quality, and child development. The evaluation involves mixed methods: quantitative caregiver surveys, digitally tracked engagement data of caregivers’ use of the digital intervention components, direct assessments of children, and focus group discussions with caregivers and key stakeholders.

**Results:**

Overall, 208 parents were recruited at baseline December 2023: 151 (72.6%) primary caregivers and 57 (27.4%) secondary caregivers. In January 2024, of these 208 parents, 168 (80.8%) enrolled in the program, which was completed in February. Postintervention data collection was completed in March 2024. Findings will be reported in the second half of 2024.

**Conclusions:**

This is the first factorial cluster randomized trial to assess the feasibility of a hybrid human-digital playful parenting program in Southeast Asia. The results will inform a large-scale optimization trial to establish the most effective, cost-effective, and scalable version of the intervention.

**Trial Registration:**

OSF Registries; https://osf.io/f32ky

**International Registered Report Identifier (IRRID):**

DERR1-10.2196/55491

## Introduction

### Background

Globally, while gains have been made in promoting access to education, the United Nations’ Sustainable Development Goal 4 and, in particular target 4.2, to ensure that, by 2030, all girls and boys have access to quality early childhood development, care, and preprimary education so that they are ready for primary education, is far from being achieved [1]. Before the COVID-19 pandemic, 43% of children in low- and middle-income countries (LMICs) were at risk of not achieving their developmental potential [2]. The COVID-19 pandemic has exacerbated this risk; between March 2020 and February 2021, an estimated 167 million preprimary-aged children had no access to early childhood education and care [3] at an age that represents a sensitive period in children’s educational development. Early childhood experiences and skills predict future academic achievement [4], with the strongest predictors being school entry–level mathematics, reading, and attention skills [5]. Furthermore, children who enter school with a knowledge of letters and numbers are likely to have achieved this because of parental instruction [6]. Thus, the *home learning environment—*the availability of educational resources (eg, books) and the quality of parenting activities (eg, reading to children and playing number games) [7]—is important for the development of foundational skills, including literacy and numeracy [8].

A World Bank review in 2023 of preschool education in Malaysia reported that preschools in the country aim to develop children’s “school readiness” for entry into compulsory primary school at age 7 years [9]. Malaysia endured one of the region’s longest periods of school closure because of the COVID-19 pandemic. Consequently, many children were poorly prepared to start primary school because of learning loss that had particularly impacted early literacy [9]. Other dimensions of children’s development, such as socioemotional and psychomotor development, were also adversely affected. Households with low incomes were disproportionately affected because state-funded preschools, that is, those in the Jabatan Kemajuan Masyarakat (Department of Community Development in Bahasa Melayu [KEMAS]), which is part of the Ministry of Rural and Regional Development) system, were closed longer than private preschools, and children from households with low incomes were less likely to have engaged in web-based schooling activities.

In addition to improving access to, and the quality of, public preschool education in Malaysia, the World Bank report recommends supporting parents’ improved involvement in preschool-aged children’s education and learning at home [9]. A national health survey in 2016 found that just 25% of children in Malaysia receive early learning stimulation at home [10]. Research has shown parenting interventions to be effective in supporting children’s learning. A meta-analysis of 14 studies in high-income countries found that parenting interventions (such as training parents to share books with their children) improved the reading development of children aged 4 to 8 years [11]. These findings have also been obtained in LMICs; for example, a randomized controlled trial of children aged 3 to 5 years in the Philippines found that, compared to the control group, children whose parents had received 1 of 3 types of training—dialogic reading coaching, early literacy skills development coaching, and numeracy skills development coaching—improved significantly in several dimensions of their development, including preliteracy skills (eg, phonological awareness and letter name knowledge) and numeracy skills [12]. This evidence reinforces the World Bank’s recommendation that parents should be supported in promoting their preschool children’s learning and development.

Preschools in Malaysia are an essential component of the country’s education system. Formal education in Malaysia typically commences at age 4 years; however, it is not compulsory. Nevertheless, most children aged <6 years are enrolled in preschool to prepare them for formal schooling and to lay the foundation for their future academic and personal development. Several agencies are involved in providing preschool education in Malaysia, including the Ministry of Education, public entities (eg, Tabika Perpaduan [Unity Kindergarten] and Taman Bimbingan Kanak-Kanak (Children’s Guidance Park [TABIKA]) KEMAS), state government–supervised institutions (eg, Majlis Agama Islam Wilayah Persekutuan [Islamic Religious Council of the Federal Territory] Islamic kindergartens and Jabatan Agama Islam Selangor [Selangor Islamic Religious Department] preschools), and private kindergartens. The early childhood education program TABIKA, organized by KEMAS, provides early education opportunities for children aged 4 to 6 years. TABIKA KEMAS preschools use a *learning through play* pedagogical approach, which aligns with children’s natural drive to play, be curious, and experiment [13].

Learning through play is essential for child development and refers to the process by which children acquire knowledge, develop skills, and explore their world through play activities. Play is a natural and instinctive behavior for children, and it serves as a powerful tool for their cognitive, social, emotional, and physical development. Positive parenting emphasizes building a strong, supportive, and nurturing relationship between parents and children. Parents can facilitate learning through play with positive parenting by providing safe and stimulating environments and play materials, allowing children to take the lead during playtime [14], and encouraging opportunities for social interactions for playing to facilitate learning [15].

Despite compelling evidence of the benefits of playful and nurturing parenting for improved learning and educational outcomes, there are significant challenges to the uptake of in-person interventions aimed at promoting such parenting [16]. In addition, local governments and service providers face multiple challenges implementing face-to-face or in-person parenting programs [17-20]. Parenting programs are often too expensive to deliver effectively at scale in low-resource settings due to their complexity, intensity, and length [21]. In Malaysia, barriers to the scale-up of in-person parenting programs include limited financing and resource allocation for such programs, and those that are implemented are usually one-off workshops with a limited or no evidence base [22].

In response to the COVID-19 pandemic and the need to create cost-effective and scalable interventions, remote-delivered parenting programs have been tested by several researchers [23]. A recent systematic review suggests positive impacts on parenting skills and childhood outcomes, although the effect may not be as great when compared to in-person–delivered programs [24]. The main barrier to implementation is difficulty in contacting parents by telephone. However, a study that used a combination of methods (SMS text messaging and group meetings) to address the restrictions associated with remote-only delivery showed an improvement in the effectiveness of the program [25].

In Malaysia, a feasibility pilot study of an in-person program highlighted the demand from parents for flexible delivery modalities [26]. Thus, a collaboration among Universiti Putra Malaysia (UPM), the University of Oxford, United Nations Children’s Fund (UNICEF) Malaysia, Malaysian government ministries, and civil society organizations has identified the need for digital and hybrid solutions to address such challenges in the scale-up of parenting programs. Web-based and hybrid delivery of such interventions has the advantage of lower costs compared to in-person–only programs, which is central to ensuring scale-up, and can potentially improve accessibility for parents who would otherwise not participate, or who have difficulty participating, in in-person programs, such as male caregivers, migrant families, and people living with disabilities.

In addition to improved learning and educational outcomes, evidence has shown parenting interventions to be effective in reducing violence against children and promoting child well-being more generally in LMICs [21,27-31]. These interventions aim to improve caregiver-child relationships through positive parenting and support parents in controlling their children’s behavioral issues with age-appropriate and effective nonviolent discipline methods. These dimensions of children’s development and welfare are particularly important in Malaysia and must also be considered. A review of the prevalence of child abuse and neglect in Malaysia reports evidence of 53% of children experiencing physical parental maltreatment, 20% experiencing emotional abuse, and 21.3% experiencing sexual abuse [32]. The impact of multitype childhood abuse is associated with negative effects on children’s psychological status (depression, anxiety, and stress) when they grow into adults [33].

In Malaysia, the Naungan Kasih (Protection through Love) Positive Parenting Program was developed in collaboration with the Malaysian government’s National Population and Family Development Board, UPM, UNICEF Malaysia, the Malaysian Association of Social Workers, Maestral International, and Parenting for Lifelong Health. National Population and Family Development Board staff members were trained to deliver the program in person to parents. Despite challenges to uptake, the intervention was effective in providing support to caregivers, improving parent-child relationships, and significantly reducing child maltreatment [26].

This study, conducted within the TABIKA KEMAS program, will assess the feasibility, acceptability, and effectiveness of ParentText delivered (1) with a WhatsApp support group and (2) with 1 or 2 in-person sessions. The results will inform a full optimization trial to determine the most effective and cost-effective version of the program, which is planned to take place across Malaysia with 800 families in the second half of 2024.

### Aims and Objectives

The primary aims of this pilot factorial cluster randomized trial are two-fold: (1) to determine the feasibility of the intervention and (2) to examine preliminary indications of the relative effectiveness of a multicomponent human-digital hybrid playful parenting intervention (ParentText combined with a WhatsApp support group and either 1 or 2 in-person sessions) at government-run preschools in Malaysia.

The objectives are as follows:

1. To determine the recruitment, retention, and engagement rates in all 4 intervention conditions and explore reasons for these rates
2. To document the extent to which the intervention was implemented in line with the program model (fidelity) in rural and periurban school settings
3. To explore the acceptability of the parenting program among caregivers, facilitators, supervisors, and members of the KEMAS leadership team
4. To estimate the costs of delivering the different intervention components
5. To provide preliminary indications of the relative effectiveness and cost-effectiveness of the different intervention components on the intended intervention outcomes, including proximal outcomes (ie, positive parenting, child physical and emotional abuse, and parent mental health and stress) and distal outcomes (ie, child learning [namely literacy, numeracy, and socioemotional development], child behavior, intimate partner relationships, financial stress, and parent quality of life).

## Methods

### Study Setting

The study will take place within the preschool system in 2 locations in Malaysia: the federal territory of Kuala Lumpur and Negeri Sembilan. Kuala Lumpur is a periurban location, while Negeri Sembilan is more rural. This contrast will provide comprehensive information pertaining to the feasibility and acceptability of the program across different contexts.

### Trial Design

This feasibility pilot is a 2×2 factorial cluster randomized trial that compares ParentText (chatbot-led parenting program) delivered to all participants, with different combinations of two engagement components: (1) a WhatsApp support group (yes or no) and (2) either 1 or 2 in-person sessions (Table 1). This study will adopt a parallel design using a 1:1:1:1 allocation ratio (each cluster at each study site exposed to 1 experimental condition).

**Table 1:** Experimental conditions from 2×2 factorial design (n=8 clusters; n=160 families).

Condition	Clusters (schools; n=8), n (%)	Caregivers (primary, n=160; primary and secondary, n=320), n (%)	Children (n=160), n (%)	Facilitators (n=8), n (%)	ParentText	WhatsApp support group (yes or no)	In-person sessions, n
1	2 (25)	40 (25); 80 (25)	40 (25)	2 (25)	On	No	1
2	2 (25)	40 (25); 80 (25)	40 (25)	2 (25)	On	Yes	1
3	2 (25)	40 (25); 80 (25)	40 (25)	2 (25)	On	No	2
4	2 (25)	40 (25); 80 (25)	40 (25)	2 (25)	On	Yes	2

### Eligibility Criteria

Study participants include caregivers, children, program facilitators, KEMAS supervisors, and members of the KEMAS leadership team. Participants must provide informed consent (parents, caregivers, program facilitators, program supervisors, and KEMAS leadership team members) or verbal assent (children) to participate in the study and before any study procedures take place.

### Inclusion Criteria

Caregivers eligible for the trial must be aged ≥18 years, be responsible for a child aged between 4 and 6 years who is registered with a KEMAS preschool, live in the same household with the child for at least 4 nights a month, and have access to a mobile phone compatible with WhatsApp.

Children eligible for the trial must be aged between 4 and 6 years, be enrolled in a KEMAS preschool, and have parental consent to participate in the study.

Teachers and facilitators of the program must be aged ≥18 years, be registered employees of a KEMAS preschool, have a diploma or certificate in early childhood education or equivalent, have participated in a facilitator training workshop, and be available to deliver the intervention package.

KEMAS supervisors must be aged ≥18 years, be registered supervisors with KEMAS, have a diploma or certificate in early childhood education or equivalent, have participated in 2 intervention program trainings (facilitator training workshop [for new facilitators to deliver the program] and training of trainers workshop [supervisors who completed the facilitator training workshop were trained to deliver training to new facilitators]).

KEMAS leadership team members must be aged ≥18 years and be federal or state administrative staff members of the Division of Early Childhood Education within KEMAS.

### Schools

Preschools from the 2 study sites were eligible for inclusion if they had sufficiently sized classes (20-25 children aged 4-6 years/class). Eight preschools were selected from a list of eligible preschools (provided by KEMAS; Multimedia Appendix 1) in Kuala Lumpur and Negeri Sembilan (4 schools per study site). Within each selected preschool, 1 class will be selected (randomly if >1 class) from which 20 families will be invited to participate in the study. Teachers of the selected classes will act as facilitators and contact the families of the children and invite 2 caregivers to participate in the study. Four children in each cluster (ie, 32 in total) will be randomly selected to participate in direct assessments.

### Intervention

The intervention is a hybrid human-digital playful parenting program Naungan Kasih*,* which consists of a chatbot-led parenting program (ParentText 2.0) delivered to all participants and two intervention engagement components: (1) a WhatsApp support group (yes or no) and (2) either 1 or 2 in-person sessions.

#### ParentText

ParentText is a chatbot-led parenting intervention that delivers personalized, gamified, scheduled, and on-demand messages through WhatsApp, audio, and visual messages to caregivers of children at different developmental stages, that is, from ages 0 to 23 months, 2 to 9 years, and 10 to 17 years. It was developed by the UK-based charities Parenting for Lifelong Health and IDEMS International. The version targeting caregivers of children aged 2 to 9 years is used in this trial, with additional content specifically relevant for caregivers of children aged 4 to 6 years.

The ParentText version used for this intervention was adapted in response to key findings from previous research in Malaysia by project partners; for example, a pre-post evaluation of the in-person version of Naungan Kasih delivered in 2 communities with low-income status between November 2018 and April 2019 found promising intervention effects on some caregiver self-reports (n=74), including reductions in overall child maltreatment, physical abuse, emotional abuse, attitudes supporting corporal punishment, and child behavior problems, as well as improvements in early childhood involvement [26]. However, no effects were found on positive parenting, harsh parenting, parental mental health, and marital satisfaction; nor were there any significant intervention effects on child (aged 10 to 17 years) reports (n=26). Qualitative findings highlighted tangible benefits for female program recipients, such as reduced use of harsh physical and verbal punishment toward their child and lower stress associated with parenting, as well as improved communications with partners [26]. However, program facilitators reported that the traveling required to facilitate in-person sessions on top of their daily work left them feeling overburdened and highlighted the potential of digital modalities.

ParentChat, a digital parenting program delivered through WhatsApp, was piloted in 2021 in Malaysia. The study found a 23% reduction in parenting stress, a 24% reduction in parental report of child behavior problems, a 15% increase in parental self-efficacy to prevent sexual abuse, and a 39% increase in intimate partner respect [34]. Caregiver attendance during the 8-week program was high; on average, parents attended 81% of the 16 sessions. In addition, a pilot of ParentText with 82 caregivers in Malaysia showed a 68% enrollment rate, with average engagement of 13 days in the chatbot. Topics most frequently accessed were *Keeping Calm*, *One-on-One Time*, and *Helping Your Children Learn*. When parents interacted with a WhatsApp message, they requested additional content provided within the chatbot 78.8% of the time. Exploratory analyses of program effects found significant improvements in positive parenting behavior. Pilot tests across Malaysia, Jamaica, the Philippines, and South Africa between 2021 and 2022 highlighted five principal areas for attention in improving ParentText [34]: (1) structural issues leading to participant dropout, (2) multimedia being insufficiently engaging, (3) the need for more and improved personalization features, (4) the need for mechanisms to promote re-engagement, and (5) issues with ease of use.

As such, the ParentText program designed for this study has been streamlined, and the goals have broadened to include those more relevant to the Malaysian preschool context; for example, content has been developed to specifically target improving learning through play and child educational outcomes. ParentText is structured around 6 goals, each with several modules (28 modules in total; Textbox 1) that take approximately 5 minutes to complete.

At program onset, caregivers choose which goal they want to start with, and each day, receive a prompt asking them whether they can complete a module related to this goal. On completion of this goal, they choose which one they would like to start next. Caregivers will have 33 days between starting and finishing the program, a time frame that allows 1 day for each module (28 days) plus 5 days to catch up on the days on which they were unable to complete a module.

Although the self-guided nature of ParentText is advantageous in terms of scalability, engagement boosters (such as social networking tools) are commonly used in parenting programs and in digital interventions more broadly to support participation [35-38]. There is evidence for the effectiveness of such engagement boosters; for example, parents taking part in a web-based version of the Triple P parenting intervention who received telephone-delivered support from practitioners engaged more with the program and reported greater program satisfaction than parents who did not receive phone support [39]. However, such improvements in engagement are not always found, as other parenting studies have documented [40]. Another option is to supplement digital interventions with in-person sessions where participants interact with program facilitators (eg, clinicians or counselors), which has been found to improve program adherence [41,42]. However, in-person sessions accrue costs associated with facilitator training [43]. This study seeks to establish whether the use of WhatsApp support groups and in-person sessions promotes engagement, satisfaction, and the effectiveness of the ParentText intervention.

**Overview of ParentText content and number of modules.**

**Goal and number of modules**

- Improve My Relationship with My Child: 5
- Support My Child’s Development: 3
- Prepare My Child for Success in School: 6
- Give My Child Structure: 5
- Support Positive Child Behavior: 4
- Keep My Child Safe and Healthy: 5

#### Component 1: WhatsApp Support Group (Yes or No)

WhatsApp support groups will be facilitated by the preschool teachers (facilitators). Each week, the teacher (facilitator) will share preformulated content on the WhatsApp group using a share button on a facilitator app. Caregivers can use the group to interact with each other and share tips, thoughts, and feelings. Facilitators will not moderate WhatsApp groups; they will only intervene if the content discussed goes against positive parenting practices.

#### Component 2: In-Person Sessions (1 or 2)

This study aims to investigate rates of engagement and acceptability for the program when participants attend 1 or 2 in-person sessions that are facilitated by preschool teachers. All participants receive the first session, which will comprise an introduction to the program and an onboarding to ParentText. Half of the participants will attend an additional second session at the end of the program, in which they will review content, role-play examples of practices they have learned, and receive a certificate of completion. It is hypothesized that this session will improve the acceptability of the program to caregivers, improve outcomes related to the content of the program thanks to the review and practice, and improve engagement by motivating parents to complete the program and receive a certificate.

### Outcomes

#### Overview

Recruitment, retention, and engagement rates (objective 1) will be measured by the indicators presented in Textbox 2.

Program fidelity (objective 2) is defined as the completion of at least 80% of facilitator-delivered components of the program. This is measured via a checklist that facilitators will complete using a dedicated app in which they will report whether they carried out planned in-person activities and whether they sent weekly WhatsApp support group messages (for those delivering this component of the intervention).

Program acceptability (objective 3) will be assessed using qualitative focus group and interview data, which will gather information from caregivers and facilitators on their views and experiences of the intervention, including what they perceive to be barriers and enablers to using it (among caregivers) and to implementing it (among facilitators). Program acceptability will also be assessed via 2 parent self-report quantitative measures (Textbox 3)***.***

**Figure 1:**
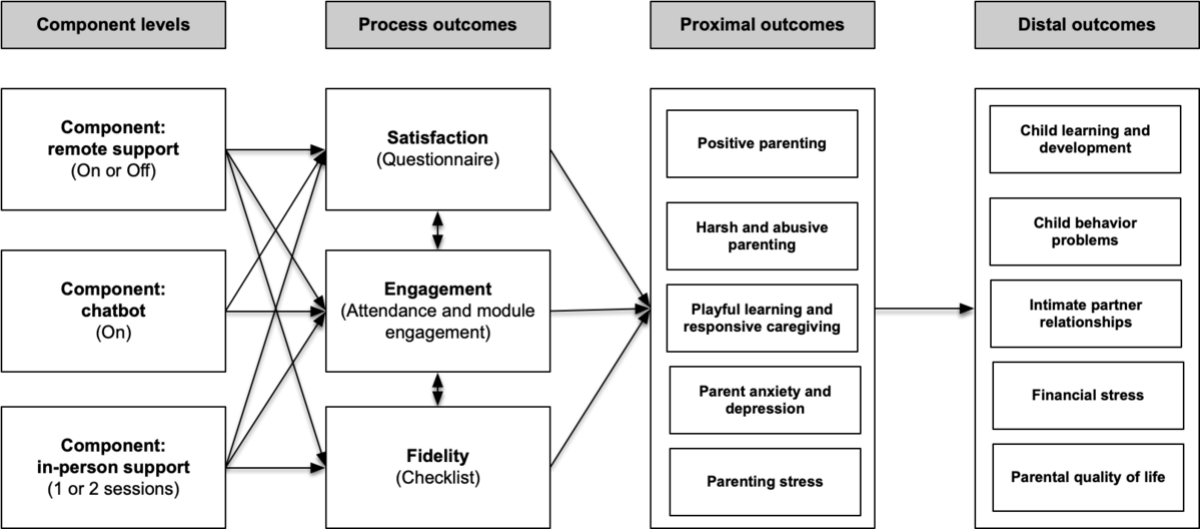
The hypothesized association among intervention components, process outcomes, and the intended primary intervention outcomes based on the theory of change conceptual model.

Figure 1 shows the hypothesized association among intervention components, process outcomes, and the intended primary intervention outcomes. Findings relevant to the aforementioned objectives will inform an understanding of the relationship between the intervention components and the process outcomes. Furthermore, these findings will facilitate an understanding of the relationship between process outcomes and the proximal outcome of positive parenting.

The cost of implementation (objective 4) will be measured from the provider perspective and will consider all resource inputs required for intervention. Cost indicators estimated will include the total cost of the intervention (set-up and operation), cost for each intervention component, and the average cost per participant enrolled (unit cost). Costs will be estimated from data gathered on actual expenditures (financial costs) and the market value of all donated and subsidized resource inputs (economic costs).

Preliminary intervention effects (objective 5) will be assessed for the main outcomes of interest, which are described in the following subsections.

**Outcomes and indicators to measure recruitment, retention, and engagement rates (objective 1).**

**Outcomes and indicators**

- Recruitment
  Number of eligible primary caregivers invited to participate and consented
  Number of eligible secondary caregivers invited to participate and consented
  Number of baseline assessments completed with primary caregivers
  Number of baseline assessments completed with secondary caregivers
  Number of child direct assessments completed at baseline
- Retention
  Number of posttest assessments completed with primary caregivers
  Number of posttest assessments completed with secondary caregivers
  Number of child direct posttest assessments completed
- Engagement
  Number of ParentText modules completed by primary caregivers
  Number of ParentText modules completed by secondary caregivers
  Number of primary caregivers who attended in-person sessions
  Number of secondary caregivers who attended in-person sessions
  Number of primary caregivers who participated in WhatsApp support groups
  Number of secondary caregivers who participated in WhatsApp support groups

**Caregiver outcomes and measures related to program acceptability (objective 3).**

**Outcomes and measures**

- Partner participation in program (postintervention assessment only)
- 8 items adapted from previous studies of Parenting for Lifelong Health interventions
- Program satisfaction (postintervention assessment only)
- 4 items adapted from previous studies of Parenting for Lifelong Health interventions

#### Proximal Outcomes

##### Harsh and Abusive Parenting

Two indicators of harsh and abusive parenting (physical and emotional abuse) will be measured using items from the International Society for the Prevention of Child Abuse and Neglect Child Abuse Screening Tool–Parent version [44]. Each item measures responses on 9-point count scales, asking the number of times a behavior occurred in the previous 2 weeks (ranging from 0=0 times to 8=8 or more times).

Child physical abuse will be measured using 4 items from the physical abuse subscale that ask questions regarding the frequency of the parents disciplining their children physically (eg, “In the last 2 weeks, how often did you discipline your child by slapping, spanking, or hitting with your hand?”).

Child emotional abuse will be measured using 5 items from the emotional abuse subscale that ask questions regarding the frequency of the parents disciplining their children emotionally (eg, “In the last 2 weeks, how often did you shout, yell, or scream at your child in an aggressive manner?”).

##### Parent-child playful learning or responsive caregiving

This will be measured using the UNICEF Multiple Indicator Cluster Surveys child development module and family care indicators [45]. The module consists of 4 items that ask parents to state the frequency of a series of learning activities on a scale ranging from 0 to 8 (eg, “How many times in the past 2 weeks (14 days) did you play with your child?”).

##### Positive Parenting

This will be measured using 2 subscales from the Parenting Young Children Scale [46], with 7 items in each scale. Parents will be asked about the frequency of these parenting practices on a scale ranging from 0=not at all to 6=most of the time during the last 2 weeks (last 14 days). The first subscale is *supporting positive behavior* (eg, “Were you able to spend time with your child in ways that were fun for both of you?”). The second subscale is *setting limits* (eg, “Were you able to stick to your rules and not change your mind?”).

##### Parent Anxiety and Depression

This will be measured using the Patient Health Questionnaire-4 [47]. This comprises 4 items relating to anxiety and depression, such as “Over the last 2 weeks, how often have you been bothered by the following problems? Feeling nervous, anxious, or on edge?” Parents are asked to respond on a 4-point Likert scale (ranging from 0=not at all to 3=nearly every day) that addresses parents’ perception of the frequency of the problems.

##### Parenting Stress

This will be measured using the Parenting Stress Scale [48], which comprises 6 items, such as “The major source of stress in my life is my child(ren),” and responses are on a 5-point Likert scale (ranging from 0=strongly disagree to 4=strongly agree) that will address the extent of agreement with the items.

#### Distal Outcomes

##### Child Learning and Development

###### Parent Report

*Child learning* or *child development* will be measured using the Measure of Development and Early Learning, a module from the Measuring Early Learning Quality and Outcomes (MELQO) framework [49]. The survey consists of 38 items asking caregivers about their child’s development across four dimensions: (1) literacy (5 items), (2) mathematics (10 items), (3) socioemotional development (21 items), and (4) executive function (2 items). Examples of items include (1) “Can [name] write his/her own name?” (literacy development), (2) “Can [name] add 3 and 2 together?” (mathematics), (3) “Does [name] share with his/her peers?” (socioemotional development), and (4) “When asked to do several things, how often does [name] remember all the instructions?” (executive function).

###### Direct Child Assessment

A subset of items from the International Development and Early Learning Assessment (IDELA) [50] will be used to measure 3 dimensions of children’s learning and development: emergent literacy (3 items), emergent numeracy (3 items), and socioemotional awareness (2 items); for example, for emergent literacy, the assessor will ask the child to identify letters in a chart; for emergent numeracy, the child will be asked to perform simple addition and subtraction tasks; and for socioemotional awareness, the child will be asked to identify the emotion being experienced by a cartoon girl who is crying.

The items chosen make up 6 of the items from the IDELA *short form*, with 2 questions related to motor skills removed, and 1 question each related to emergent literacy and emergent numeracy added. This was decided because of the ParentText intervention’s greater prioritization of these dimensions of children’s early learning with respect to motor skills.

###### Child Behavior

*Externalizing problems* of the child will be measured using the Child and Adolescent Behavior Inventory [51]. The scale consists of 12 items asking parents about their child’s behavior, such as physical aggression, defiance, theft, and vandalism, in the past 2 weeks. Examples of items are “He/she is quick-tempered and has fits of anger” and “He/she destroys things.” Responses are based on a 3-point Likert scale (ranging from 0=not true to 2=very true).

##### Intimate Partner Relationships

###### Gender-Equitable Behaviors

Four items will assess the frequency of a selection of gender-equitable behaviors; for example, “In the past 2 weeks, how often did you and your partner share housework and caregiving tasks equally?” Responses are based on a 5-point Likert scale (ranging from 1=never to 5=most of the time).

###### Marital Health Quality

The Marital Health Scale [52] will be used to assess the marital health of the parents. The 5-item scale will ask parents about the extent of agreement in regard to 5 questions about their marriage. Each item will include a similar theme (in parentheses) for the parents to consider when providing answers regarding their marriage (eg, “My marriage is satisfactory” [content, fulfilled, gratified]). Responses will be based on a 5-point Likert scale (ranging from 1=strongly disagree to 5=strongly agree).

###### Financial Stress

This will be measured using the Financial Self-Efficacy Scale [53], which consists of 6 items that ask about financial behavior (eg, “It is hard to stick to my spending plan when unexpected expenses arise” and “I lack confidence in my ability to manage my finances”). Responses are based on a 4-point Likert scale.

###### Parents’ Health-Related Quality of Life

The Assessment of Quality of Life-4D instrument [54] will be used to evaluate the parents’ health-related quality of life, which will be measured to facilitate an economic evaluation of the program’s impact on quality-adjusted life years. There are 12 items that ask questions related to dimensions such as sleep, relationships, and pain, and responses are chosen on a 4-point Likert scale (eg, “Because of your health, your relationships [for example: with your friends, partner or parents] generally: [responses range from] Are very close and warm [to] I have no close and warm relationships”).

###### Partner Participation in Program

This will be measured at postintervention assessment only. Primary caregivers will be asked 8 questions about their partner’s participation or nonparticipation in the program (eg, “If your partner or spouse participated in the program, did s/he share with you what they had learnt in the training?”). Items will be measured on a 3-point Likert scale.

###### Program Satisfaction

This will be measured after the intervention only. Caregivers will be asked 4 questions related to their satisfaction with the program on a 5-point Likert scale (eg, “Would you recommend the program to a friend or relative?” Responses range from 1=strongly not recommend to 5=strongly recommend).

Textbox 4 presents a summary of the intervention effectiveness outcomes, along with measures and number of items.

**Summary of intervention effectiveness outcomes.**

**Outcomes and measures**

- Proximal outcomes
- Child physical abuse: International Society for the Prevention of Child Abuse and Neglect (ISPCAN) Child Abuse Screening Tool–Trial, physical abuse subscale (4 items)
  Child emotional abuse: ISPCAN Child Abuse Screening Tool–Parent version, emotional abuse subscale (5 items)
  Playful learning: United Nations Children’s Fund Multiple Indicator Cluster Surveys child development module and family care indicators (4 items)
  Parenting: Parenting Young Children Scale (supporting positive behavior, and setting limits; 14 items)
  Parent mental health: Patient Health Questionnaire-4 (4 items)
  Parenting stress: Parenting Stress Scale (6 items)
- Distal outcomes
- Child learning and development: MELQO framework’s Measure of Development and Early Learning module teacher or caregiver report item (38 items); IDELA (8 overarching items, total 21 subitems)
- Child behavior—externalizing problems: Child and Adolescent Behavior Inventory, externalizing subscale (12 items)
- Gender-equitable behaviors: adapted from questionnaires used in previous violence prevention studies (4 items)
- Marital health or quality: Marital Health Scale (5 items)
- Financial stress: Financial Self-Efficacy Scale (5 items)
- Quality of life: Assessment of Quality of Life-4D (12 items)

#### Demographic Information

Caregiver and child demographic information will be collected at baseline and include caregiver age, marital status, employment, educational attainment, literacy, and household socioeconomic status; child age; family health (eg, parent and child disability) and family vulnerabilities (eg, substance abuse by household member); child’s relationship to caregiver; presence of child’s biological parents and other child in the house; and presence of another person who shares the responsibility of child rearing and their relationship with the caregiver.

#### Intervention Effectiveness

Findings related to intervention effectiveness will inform the extent to which intervention components and their related process outcomes are associated with the primary proximal outcome of positive parenting, the primary distal outcome of child development, and other outcomes, namely child maltreatment, child behavior problems, and parent mental health.

**Figure 2:**
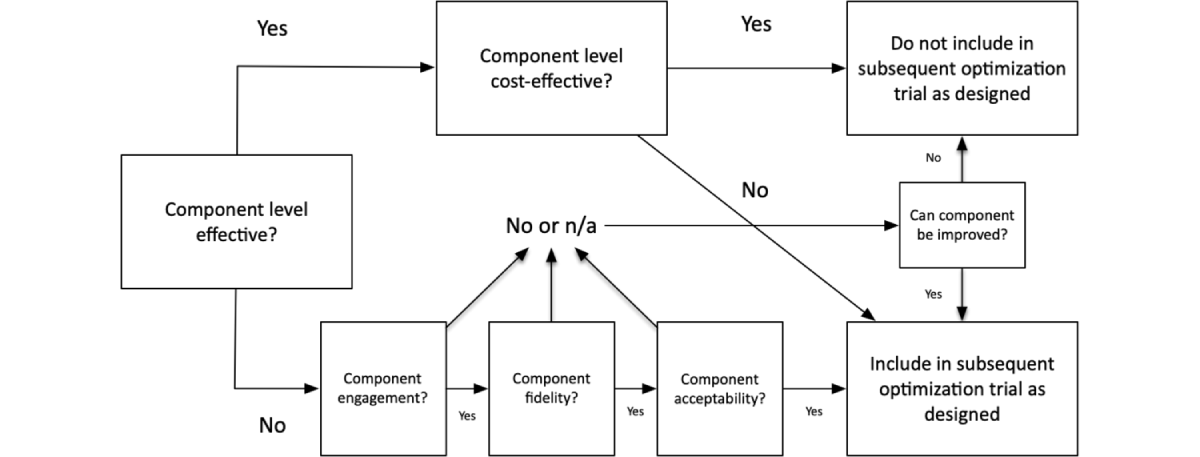
Decision-making model to determine the inclusion of intervention components in a full trial. N/A: not applicable.

Overall, findings related to the 5 study objectives will inform decision-making regarding whether to include intervention components or which level of intervention component to include in future evaluations of the optimized version of the Naungan Kasih program. This is illustrated in Figure 2. Thus, if a component level is both effective and cost-effective, it will not be included in a subsequent optimization trial. If it is effective but not cost-effective, it will be evaluated in a subsequent optimization trial. If it is not effective but is feasible (ie, there is engagement, it is acceptable, and it is implemented with fidelity), it will be included in a subsequent optimization trial. If it is not effective and not feasible but can be improved, the improved version will be evaluated in a subsequent optimization trial. If it is not effective, not feasible, and cannot be improved, then it will not be included in a subsequent optimization trial.

#### Participant Timeline

The participant timeline is summarized in Table 2. Parents invited to participate by facilitators will be asked to attend an in-person session that will include (1) eligibility screening, (2) informed consent, and (3) baseline assessment. The baseline sessions took place in schools from December 6 to 16, 2023. Schools were then randomly allocated to the 4 treatment conditions. The intervention began with onboarding sessions that took place in schools from January 4 to 6, 2024. The intervention ran until February 7, 2024. Qualitative assessments with facilitators, supervisors, and KEMAS leadership team members were conducted between February 5 and 16, 2024. Posttest data collection was carried out with parents and children between February 24 and March 12, 2024.

**Table 2:** The SPIRIT (Standard Protocol Items: Recommendations for Interventional Trials) schedule for participant enrollment, intervention, and assessments. FGD: focus group discussion; KEMAS: Jabatan Kemajuan Masyarakat (Department of Community Development).

		Study period			
		Enrollment and baseline (time point 1)	Allocation	After allocation (intervention)	After the intervention (time point 1)
**Enrollment**					
	Eligibility screen	✓			
	Informed consent	✓			
	Allocation		✓		
**Interventions**					
	Condition 1			✓	
	Condition 2			✓	
	Condition 3			✓	
	Condition 4			✓	
**Assessments**					
	Recruitment	✓			✓
	Retention				
	Adherence			✓	
	Engagement			✓	
**Parent survey**					
	Recruitment	✓			✓
	Demographics	✓			
	Child physical abuse	✓			✓
	Child emotional abuse	✓			✓
	Playful learning	✓			✓
	Child learning and development (parent report)	✓			✓
	Child behavior—externalizing problems	✓			✓
	Parenting	✓			✓
	Parent mental health	✓			✓
	Parenting stress	✓			✓
	Gender-equitable behaviors	✓			✓
	Marital health	✓			✓
	Financial stress	✓			✓
	Quality of life	✓			✓
	Partner participation in program	✓			✓
	Program satisfaction	✓			✓
Child development and learning (child direct assessment)		✓			✓
Costs				✓	
**Qualitative assessments**					
	Parent FGDs^a^				✓
	Facilitator interviews				✓
	Supervisor FGD				✓
	KEMAS^b^ leadership FGD				✓

^a^FGD: focus group discussion.

^b^KEMAS: Jabatan Kemajuan Masyarakat (Department of Community Development).

#### Sample Size

A formal sample size calculation was not used for this feasibility pilot study. It is anticipated that the sample size of 160 families (160 primary caregivers and up to 160 secondary caregivers, as well as 160 children from the same households) split equally across 8 preschools (4 at each study site) will provide sufficient information on the study objectives to inform a future main trial. In 20% (32/160) of the families at each preschool, direct assessments will be conducted with the child. The 8 project facilitators will be invited to participate in interviews. Five supervisors will be invited to participate in a focus group discussion (FGD). Finally, 5 KEMAS leadership team members will be invited to participate in an FGD.

#### Recruitment

Recruitment of KEMAS leadership team members, supervisors, and facilitators took place between July 4 and September 15, 2023. Supervisors and facilitators took part in the Naungan Kasih training during the second week of November 2023.

##### KEMAS Leadership Team Members

UPM used existing contacts with KEMAS to invite members to participate in the study. KEMAS leadership team members were sent an information sheet and consent form via email.

##### KEMAS Supervisors

Members of the KEMAS leadership team identified and invited school supervisors in the KEMAS system to confirm their willingness to participate in a training of trainers session.

##### Facilitators

Members from the KEMAS leadership team identified and invited teachers at the selected preschools to confirm their willingness to participate as program facilitators.

##### Caregivers

Facilitators (class teachers) informed parents at the selected preschools about the study in mid-November 2023, after the training program. Two parents from each household have been invited to attend the registration and baseline data collection session. During the in-person data collection session, research assistants will screen the attendees to confirm their eligibility and establish which parent is the primary caregiver and which one the secondary caregiver. The research assistants will then ask parents to provide full written consent and complete the baseline assessment. Caregivers who do not attend the in-person data collection session but who indicated their interest in participating will be contacted by UPM researchers in the 2 weeks after the in-person session. The researcher will screen each caregiver to confirm their eligibility and establish which parent is the primary caregiver and which one the secondary caregiver. The researcher will then ask them to provide full verbal consent and carry out the baseline survey with them verbally over the telephone. Parents who provide consent and who complete the baseline assessment will be invited to participate in the first in-person session of the intervention, the onboarding session.

##### Children

A random subsample of 4 children from each cluster will be selected to participate in direct assessments. Primary caregivers attending the in-person baseline data collection session will be asked to provide written consent to their child’s participation. The parent will then select a bead from a bag to determine the random selection of the child. For the children selected, the assessment will take place at the school during the 2 weeks after the parents’ in-person informed consent and baseline data collection session. The children selected will be asked to provide verbal assent.

#### Assignment of the Intervention

##### Allocation

###### Sequence Generation

Within each study site, schools will be randomly allocated in a 1:1:1:1 ratio to 1 of the 4 treatment conditions (described in the Trial Design subsection) using a computerized random number function in Microsoft Excel. The list of schools will be sorted in Excel by study site (strata) and then by random number, and within each stratum, schools will be manually assigned to the 4 experimental conditions: the school with the lowest random number will be assigned to experimental condition 1, the school with the second lowest random number to experimental condition 2, and so on.

###### Allocation-Concealment Mechanism

Randomization will be conducted after the final baseline data collection session. Data collectors, facilitators, and participants will be concealed to allocation until the start of the intervention. The onboarding session will take place between the final data collection session and the start of the intervention, at which point the facilitators and the intervention recipients will be informed about condition allocation. Condition allocation will remain concealed throughout the study for data collectors. Those involved in analysis will remain blinded until main analyses have been conducted; however, they may become aware of condition allocation when analyzing FGD data because of particular questions and answers related to components of the study relevant to the intervention conditions.

##### Implementation

Before randomization, a list of recruited schools will be sent to the University of Oxford by UPM researchers. The data manager, based at the University of Oxford, will then randomly assign schools to the experimental conditions. The randomization sequence will be in a password-protected file and accessed on a need-to-know basis. After randomization, the allocation of each school will be forwarded to UPM researchers, who will share this with the implementing facilitators at the relevant schools.

##### Blinding

During baseline assessment, UPM researchers and intervention participants will be blinded to condition allocation and will not be informed about the range of conditions and the allocation status of other schools to reduce the risk of contamination. During the intervention onboarding process, participants will be exposed to information pertaining to the condition to which they are assigned. Blinding will not be possible for the facilitators because of their involvement in program implementation. UPM research assistants will be blinded to the allocation of schools during the postintervention assessments. FGDs will take place after the collection of postintervention data. It is expected that research assistants will learn about the allocation of clusters, as will the teacher-facilitators, during the FGDs. Statisticians will be blinded to cluster and participant allocation when carrying out quantitative analyses.

#### Data Collection, Management, and Analysis

##### Data Collection

This feasibility pilot study will use a mixed methods approach using both quantitative and qualitative data.

###### Quantitative Data

These data will include digitally tracked engagement data, facilitator administrative data and self-reports on program implementation, caregiver baseline and posttest surveys, and direct assessments of children.

Digitally tracked data on parent engagement with the chatbot will be collected automatically through parent interactions with ParentText. These data will be linked to the individual participant ID. Facilitator administrative data will include a register of caregivers invited to participate in the intervention and whether they consented to participate. Facilitators will record caregiver participation in the WhatsApp support groups via the facilitator app. Information recorded will include whether caregivers sent a message (participated or did not participate) in the WhatsApp group over the week during which facilitation content messages were sent. Finally, facilitators will also complete checklists on the activities they carried out in in-person sessions and whether they shared program content on the WhatsApp support groups.

Caregiver self-completion assessments will be administered at 2 time points: baseline and after the intervention. A longer survey will be administered to primary caregivers, and a shorter version will be administered to secondary caregivers. Baseline data were collected between December 6 and 16, 2023. Postintervention data will be collected from all participants 3 weeks after the second in-person session (for participants receiving 2 in-person sessions); all other participants (those receiving only 1 in-person session) will be invited to the school to complete the assessment. All assessment data will be collected using Open Data Kit (ODK; Get ODK Inc). UPM researchers will provide assistance to caregivers with low digital literacy. Caregivers consenting to participate in ParentText but who are unable to attend the in-person session will be contacted by UPM researchers who will administer the survey over the telephone after informed consent and enter responses using ODK on tablet computers.

Direct assessments of a subsample of children (32/160, 20%) will be undertaken at baseline and after the intervention by 2 UPM researchers from the field of child development who will enter scores on the observational measure into ODK on tablet computers.

###### Qualitative Data

These data will include FGDs with caregivers, KEMAS supervisors, and KEMAS leadership team members as well as individual interviews with facilitators. All FGDs and interviews will be conducted by qualitative researchers from UPM. Participants will be asked for their consent to the discussions and interviews being audio recorded. If any participants decline, notes will be taken instead.

FGDs among caregivers will be conducted at 4 randomly selected schools, and each school will represent 1 condition. Caregivers from the selected schools will be invited to participate with other caregivers from the same school. Two FGDs for each class (1 FGD with male participants and 1 FGD with female participants) will be conducted, resulting in 8 FGDs.

In-depth interviews with 8 facilitators will be held in the 2 weeks after caregivers’ completion of the intervention. Interviews will be conducted via web-based videoconferencing.

KEMAS supervisors will participate in an FGD that will be held via web-based video conferencing. KEMAS leadership team members will also participate in an FGD that will be held via web-based videoconferencing. These 2 FGDs will be held during the week after the interviews with the teacher-facilitators.

##### Data Management

Survey data collection will be carried out through computer-assisted self-interviews using tablet computers, which will be stored in a locked cabinet at the UPM research office and accessed only by authorized personnel on data collection dates. All data collected on tablet computers and exported to ODK will be encrypted as soon as the survey is completed by the interviewer and will be accessible only to senior research personnel on a need-to-know basis. Each tablet computer will have a GPS tracking application that will permit remote deletion of stored information in the event of theft.

Paper-based surveys will be used in cases where tablet computers malfunction. These surveys will also be stored in a locked cabinet at the UPM research office. They will be destroyed once data have been transferred to ODK via tablet computers and verified for accuracy by the research team.

Quantitative data from the baseline and postintervention assessments will be anonymized and stored in a password-protected University of Oxford secure server. Data on this server will be managed by the University of Oxford central IT team and the Global Parenting Initiative (GPI) data management team, with support from the Department of Social Policy and Intervention IT office. Access, which will be managed by the GPI data management team, will be restricted to study team members or partner organizations involved in the research. Engagement data from ParentText will be stored using end-to-end encryption on the UNICEF server. Participants will be assigned a unique ID number during data collection that will enable linkage of the data across data sets.

Audio recorders will be used for in-person FGDs. Web-based FGDs and interviews will be recorded using the Google Meet audio recording function. Audio recordings from the FGDs and interviews will be transcribed verbatim and stored on password-protected devices, then securely uploaded and backed up on the University of Oxford server. Subsequently, all audio files will be deleted from the recording devices. Transcripts will be anonymized and verified, after which audio recordings will be permanently deleted from the databases.

All electronic documents will be named following a standard format, including the date as version control. Access to anonymized transcripts will be granted only by the GPI data manager via OneDrive for Business (Microsoft Corp) at the University of Oxford. Anonymized data sets will be stored securely for perpetuity and use per UK Data Archive guidance [55]. Raw data will be owned by the University of Oxford and UPM and stored by the University of Oxford. External access to the data will require approval from both principal investigators.

Data cleaning will be conducted during data collection to monitor data quality. Individual raw data sets from the baseline and postintervention evaluations will be stored separately from the final merged data set so that data reference points are available in the data validation process. All electronic documents and data, including quantitative data and transcripts of qualitative data, will be stored on at least 2 servers with access granted individually on a need-to-know basis. Thus, data will be protected from both server failure and confidentiality breaches.

##### Statistical Methods

All statistical analyses will be performed using Stata (StataCorp LLC) or R statistical software (R Foundation for Statistical Computing). Summary statistics will be used to describe all variables of interest. Descriptive analysis will be conducted to analyze recruitment, retention, adherence, and engagement data (objective 1), and all statistical tests will be 2-tailed with α=.05.

Intervention effects on primary and secondary outcomes (objective 5) will be represented by point estimates and their SEs. The main effect for each intervention component on primary and secondary outcomes will be estimated using multilevel models (including mixed models where outcomes are continuous, Poisson models where outcomes are counts or count distributed, and logistic models where outcomes are binary). Each model will specify 3 levels to account for the longitudinal and clustered nature of the data: repeated measures are nested within individuals, which are, in turn, nested within schools. Level 1 will include a term for categorical time (before and after the intervention) and for the interaction between time and intervention status; level 2 will include terms for individual sociodemographics such as age (parent and child) and gender (parent) and other individual-level covariates, centered at the sample mean; and level 3 will include terms for the intervention components and the stratifying factor. Robust SEs will be estimated to adjust for clustering. This study will report the direction and magnitude of standardized betas, incidence risk ratios, and odds ratios at a significance level of *P*<.10 with 90% CIs. Two-tailed tests will be conducted across all analyses.

Intervention costs (objective 4) will be estimated from the provider perspective. Resource inputs valued to estimate costs will include facilitator costs, such as those associated with their training to deliver ParentText (captured as their time spent attending training sessions) and with preparing and delivering specific intervention components; physical space used to deliver in-person sessions, which will be audited and valued; and travel and supplies (such as internet data and mobile phones). Local researchers and coordinators will collect resource use data in real time (ie, alongside the full study trial) via the completion of weekly ODK-based surveys. Costs related to the development of ParentText and content adaptation and translation will be included as a capital start-up cost. As the cost analyses will be conducted from the provider perspective, costs incurred by participants (travel expenditure and opportunity costs) will be excluded. While the implementing organization’s program monitoring and evaluation costs will be included in the cost estimates, broader research activity costs will be excluded. Program implementation costs and outcomes data will be recorded and analyzed in Excel. The total cost for each intervention condition will then be compared against the number of participants who completed all ParentText modules and for the positive parenting outcome.

Regarding qualitative data, the audio recordings from the interviews and FGDs will be transcribed by UPM researchers. Qualitative data will be analyzed in Bahasa Melayu using NVivo (Lumivero) and Microsoft Word using framework analysis. The analysis will include five stages: (1) familiarization, (2) the identification of themes, (3) indexing, (4) charting and summarization, and (5) interpretation and mapping. Researchers will begin by familiarizing themselves with the transcripts and identifying emerging themes. A code will then be assigned to each theme and subtheme at the indexing stage. After themes are indexed, they will be charted and summarized. Finally, interpretation and mapping will be used to develop a deeper understanding of the findings.

#### Data Monitoring

UPM and University of Oxford research teams will oversee the study procedures, including implementation, participant safety, and study conduct. The research teams will also monitor data management throughout its duration, including baseline and postintervention data collection, cleaning, and storage and analysis, and ensure the quality and rigor of the study conduct. A survey monitoring checklist will be created to keep track of errors in data quality checks, such as incorrectly entered names in the participant ID list and duplicates in the data.

#### Harms

This pilot study will carry out safeguarding strategies developed from universal principles of ethics, respect, beneficence, and justice. The local UPM research team will be trained on the safety protocol for this study. UPM also has its own guidelines on protection from sexual exploitation and abuse (PSEA) under the policy of zero tolerance toward sexual harassment. The guidelines define UPM’s commitment to PSEA with regard to adults and children considered vulnerable. The PSEA guidelines fulfill the requirements of the United Nations in ensuring adequate safeguards and action related to PSEA.

Caregivers will be informed of any risks related to their participation in the study. They will have the right to decline any assessment and participation at any time. Caregivers can opt out of ParentText at any time by typing “STOP MESSAGES” in the ParentText WhatsApp chat. Any withdrawal from the study will not affect participants’ rights to other services or result in any penalty.

#### Ethical Considerations

Ethics approval for this study was granted by the University of Oxford Research Ethics Committee (R88954/RE001) on September 18, 2023, and by the UPM Ethics Committee for Research Involving Human Subjects (JKEUPM-2023-1226) on October 15, 2023.

#### Consent or Assent

##### Caregivers and Children

Trained program facilitators will collect informed consent from adults and informed assent from children before baseline assessment at local schools in the communities where the study is taking place. Informed consent and assent forms will include clear descriptions of the intervention; study objectives; the use of, and protection measures for, participant data; and participants’ rights to refuse to respond to survey questions or withdraw at any point from the study. Children’s participation will be conditional on their parents providing consent and their providing verbal assent. Adults who agree to participate in the intervention will be invited to sign a paper-based informed consent form to indicate their consent.

##### KEMAS Leadership Team Members

UPM researchers will contact potential participants by telephone and read out the information sheet and consent form. If they agree to participate, they will be asked to sign the consent form and email it to the UPM research coordinator, who will print and securely store the form and delete the email.

##### KEMAS Supervisors

UPM researchers will obtain informed consent from supervisors after they attend an in-person training of trainers session. UPM researchers will read out the study information and respond to any questions or concerns that participants raise. Supervisors who agree to participate will be asked to sign a paper-based informed consent form. Those who sign will be invited to participate in an FGD after the completion of the program.

##### Facilitators

UPM researchers will obtain informed consent from teachers after they attend the Naungan Kasih training session. UPM researchers will read out the study information and respond to any questions or concerns that participants raise. Teachers who agree to participate will be asked to sign a paper-based informed consent form. Those who sign will be invited to participate in a qualitative interview after the completion of the program.

#### Privacy and Confidentiality

Anonymized baseline and postintervention data sets will be stored in a password-protected server at the University of Oxford. Access will be controlled and only granted to members of the study team or partner institutions that aided in the research project. Audio recordings of the interviews and FGDs will be transcribed verbatim and stored in password-protected devices and will then be securely uploaded and backed up at the University of Oxford server. Once uploaded to the server, the audio files will be deleted from the recording devices. After the transcripts have been anonymized and verified, the audio recordings will be permanently deleted from data repositories. All Excel and Word files will be named following a standardized protocol, including the download date, to ease version control. Deidentified interview transcripts and data sets will only be shared by the GPI data manager by granting access to specific files through OneDrive for Business at the University of Oxford. Deidentified data sets will be stored securely for perpetuity and use per UK Data Archive guidance. Raw baseline and postintervention data collected will be owned by UPM and the University of Oxford.

Data captured from ParentText is stored in an encrypted anonymous database managed by UNICEF Malaysia. Personally identifiable information, such as the user’s telephone number, is never stored. Data captured from the facilitator app are also anonymous and stored in an encrypted server hosted by IDEMS International. Both the application and chatbot are General Data Protection Regulation compliant.

#### Compensation

Parents will receive compensation for their participation at 3 stages of the research: survey completion at baseline data collection, survey completion at postintervention data collection, and participation in FGDs at postintervention data collection. They will receive MYR 50 (US $10.5) for their participation at each stage. They can therefore receive up to MYR 150 (US $31.5) for participation in the study.

#### Access to Data

Only the University of Oxford and UPM will have access to the raw research data. Access to clean and pseudoanonymized data sets and interview transcripts will be managed by the GPI data manager and evaluated on a need-to-know basis. UNICEF and the Malaysian Association of Social Workers, the implementing partners, will also have access to these data sets to assist with data quality checks.

#### Ancillary and Posttrial Care

Safeguarding procedures will be used to mitigate risks to participants. The informed consent form for study participants indicates that if the participant feels anxious or worried during their involvement in the trial, they will be offered support such as counseling.

## Results

Recruitment began on December 6, 2023, and as of December 29, 2023, a total of 208 caregivers had been recruited into the trial. The intervention was completed on February 7, 2024. Follow-up data collection was completed on March 12, 2024. Data management is still in progress; therefore, data analysis has yet to be performed. Results are expected to be published in the second half of 2024.

## Discussion

### Summary

This factorial cluster randomized trial is the first to evaluate the feasibility of a hybrid human-digital playful parenting program in Southeast Asia. It explores this in 2 contrasting contexts in Malaysia—an urban setting in Kuala Lumpur and a rural setting in Negeri Sembilan—which aims to maximize the generalizability of its results. The findings from this study will contribute to understanding the viability for such a hybrid program to be scaled up in similar contexts to Malaysia. By examining the feasibility, acceptability, and effectiveness of ParentText, which is delivered along with a WhatsApp support group and 1 or 2 in-person sessions, the study will provide important insights into the optimal combination of intervention components. The findings may identify versions of the intervention that are considerably more or less feasible than others, which could inform their inclusion or exclusion in future evaluations of the program.

In addition to this trial, other trials of different versions of ParentText are currently underway that will further contribute to the evidence base on the feasibility and effectiveness of hybrid parenting programs. In South Africa and Tanzania, cluster randomized controlled trials are being conducted among caregivers of adolescent girls (in South Africa) [56] and caregivers of adolescents (in Tanzania) [36,57].

### Limitations

This study has 2 main limitations. First, because the aim of the study is primarily to evaluate feasibility and not effectiveness, the sample size is likely too small to have the statistical power to detect significant differences in outcomes among the different program versions. Second, the study will not provide information on the reasons for nonparticipation of those who did not enroll. Given that this study aims to investigate the acceptability of the program, understanding barriers to participation would inform potential adaptations to its content and delivery in preparation for future testing to maximize recruitment and engagement.

## Appendix

Multimedia Appendix 1 List of Jabatan Kemajuan Masyarakat (Department of Community Development; KEMAS) preschools for the feasibility pilot study.

## Abbreviations

FGD: focus group discussion
GPI: Global Parenting Initiative
KEMAS: Jabatan Kemajuan Masyarakat (Department of Community Development)
LMIC: low- and middle-income country
ODK: Open Data Kit
PSEA: protection from sexual exploitation and abuse
TABIKA: Taman Bimbingan Kanak-Kanak (Children’s Guidance Park)
UNICEF: United Nations Children’s Fund
UPM: Universiti Putra Malaysia

## Data Availability

The data sets generated during and analyzed during this study will be available in the OSF repository [58].
