# Supplementary material for: A Hybrid Digital Parenting Program Delivered Within the Malaysian Preschool System: Protocol for a Feasibility Study of a Small-Scale Factorial Cluster Randomized Trial
Source: JMIR Res Protoc. 2024 Apr 26;13:e55491. doi: 10.2196/55491 (PMC11087859; doi:10.2196/55491)
Supplement: Multimedia Appendix 1 [file resprot_v13i1e55491_app1.docx]

Attachment 1. List of KEMAS preschools for the feasibility pilot study.

| **No** | **State** | **School name & address** |
| --- | --- | --- |
| 1 | Wilayah Persekutuan Kuala Lumpur | TABIKA KEMAS  PPR SERI MALAYSIA (KELAS 1) |
|  |  | BLOK B-00 05 JALAN 1/125 A,  TAMAN DESA PETALING, 57100 KUALA LUMPUR |
| 2 |  | TABIKA KEMAS  PPR INTAN BAIDURI (KELAS 2) |
|  |  | PUSAT KOMUNITI PPR INTAN BAIDURI, PPR INTAN BAIDURI, 52100 TAMAN INTAN BAIDURI |
| 3 |  | TABIKA KEMAS  PPR KG MUHIBBAH (KELAS 3) |
|  |  | JALAN 2/155 OFF JALAN PUCHONG,  58200 KUALA LUMPUR |
| 4 |  | TABIKA KEMAS  PINGGIRAN BUKIT JALIL D |
|  |  | BLOK D-00-02 & 03, PPR PINGGIRAN BUKIT JALIL  TAMAN BUKIT JALIL INDAH, 58000 KUALA LUMPUR |
| 5 | Negeri Sembilan | TABIKA KEMAS  KG NYATOH |
|  |  | TABIKA KEMAS KG NYATOH, 72100 RANTAU NEGERI SEMBILAN 72100 SEREMBAN |
| 6 |  | TABIKA KEMAS  TAMAN SEREMBAN JAYA 3 |
|  |  | JALAN 3/2A TAMAN SEREMBAN JAYA,  70450 SEREMBAN |
| 7 |  | TABIKA KEMAS  TAMAN DESA CEMPAKA |
|  |  | PT11166 JLN BBN 6/1 G, TAMAN DESA CEMPAKA,  71800 NILAI |
| 8 |  | TABIKA KEMAS  KG LUBOK CHINA |
|  |  | 71150 LINGGI NEGERI SEMBILAN |
